# Congenital central hypoventilation syndrome in korea: 20 years of clinical observation and evaluation of the ventilation strategy in a single center

**DOI:** 10.1007/s00431-024-05611-6

**Published:** 2024-05-23

**Authors:** Min Jeong Lee, Ji Soo Park, Kyunghoon Kim, Jung Min Ko, June Dong Park, Dong In Suh

**Affiliations:** 1 Department of Pediatrics, Seoul National University College of Medicine 101, Daehak-Ro, Jongno-Gu, Seoul, 03080 Republic of Korea; 2 Department of Pediatrics, Seoul National University Bundang Hospital, Seongnam, Republic of Korea

**Keywords:** Autonomic dysfunction, CCHS, Neurodevelopmental delay, PHOX2B

## Abstract

Congenital central hypoventilation syndrome (CCHS) is a rare genetic disorder characterized by hypoventilation due to impaired breathing control by the central nervous system and other symptoms of autonomic dysfunction. Mutations in paired-like homeobox 2 B (*PHOX2B*) are responsible for most cases of CCHS. Patients with CCHS have various phenotypes and severities, making the diagnosis difficult. This study aimed to present a comprehensive single-center experience of patients with CCHS, including key clinical features, treatment strategies, and outcomes. A retrospective chart review was performed for patients diagnosed with CCHS between January 2001 and July 2023 at Seoul National University Children’s Hospital. Finally, we selected 24 patients and collected their demographic data, genotypes, ventilation methods, and clinical features related to autonomic dysfunction. The relationship between the clinical manifestations and genotypes was also examined. All patients used home ventilators, and tracheostomy was performed in 87.5% of patients. Fifteen (62.5%) patients had constipation and nine (37.5%) were diagnosed with Hirschsprung disease. Arrhythmia, endocrine dysfunction, and subclinical hypothyroidism were present in nine (37.5%), six patients (25.0%), and two patients (16.7%), respectively. A significant number of patients exhibited neurodevelopmental delays (19 patients, 79.2%). There was a correlation between the phenotype and genotype of *PHOX2B* in patients with CCHS. (r = 0.71, p < 0.001).

*Conclusion*: There was a positive correlation between paired-like homeobox 2 B mutations (especially the number of GCN repeats in the polyalanine repeat mutations sequence) and clinical manifestations. This study also demonstrated how initial treatment for hypoventilation affects neurodevelopmental outcomes in patients with CCHS.

**Table:** 

**What is Known:** *• Congenital central hypoventilation syndrome is a rare genetic disorder characterized by hypoventilation and dysfunction of autonomic nervous system.* *• The disease-defining gene of CCHS is* *PHOX2B* *gene – most of the cases have heterozygous PARMs and the number of GCN triplets varies among the patients(20/24 – 20/33).*	
**What is New:** *• We have noted in the Korean patients with CCHS that there is a correlation between genotype (number of GCN repeats) and severity of phenotype.* *• National support for rare diseases allowed for a prompter diagnosis of patients with CCHS in Korean population.*

**What is Known:**

*• Congenital central hypoventilation syndrome is a rare genetic disorder characterized by hypoventilation and dysfunction of autonomic nervous system.*

*• The disease-defining gene of CCHS is* *PHOX2B* *gene – most of the cases have heterozygous PARMs and the number of GCN triplets varies among the patients(20/24 – 20/33).*

**What is New:**

*• We have noted in the Korean patients with CCHS that there is a correlation between genotype (number of GCN repeats) and severity of phenotype.*

*• National support for rare diseases allowed for a prompter diagnosis of patients with CCHS in Korean population.*

## Introduction

Congenital central hypoventilation syndrome (CCHS) is a rare genetic disorder characterized by hypoventilation, particularly during sleep, due to impaired central control of breathing and autonomic dysfunction. While the incidence of CCHS has been reported to be 1 case per 148,000 live births in Japan and 1 case per 200,000 live births in France [1, 2] recent incidence of CCHS in Korea is estimated to be 1/52,000–1/90,000 live births [3]. Clinical hallmarks of CCHS include central hypoventilation and sleep apnea due to reduced or absent ventilatory responses to hypercapnia and hypoxia and associated autonomic nervous system dysfunction (ANSD) manifestations, such as Hirschsprung disease (HD), cardiac arrhythmia, and neural crest-derived tumors [4]. Most cases present with clinical symptoms in the neonatal period due to hypoventilation; however, later-onset presentations are increasingly recognized during childhood and adulthood [4].

Paired-like homeobox 2B (*PHOX2B*) is a disease-defining gene in patients with CCHS. Associated *PHOX2B* mutations are mostly related to the expansion of GCN triplets in the 20 polyalanine coding regions in exon 3, defined as polyalanine repeat mutations (PARMs). Most cases had heterozygous PARMs for alanine residues 24–33, with genotypes expressed as 20/24–20/33. A minority of patients with CCHS have a non-PARM (NPARM) mutation, in which the gene is mutated outside the PARM frame, including frameshift, nonsense, and missense mutations of *PHOX2B*. A genotype–phenotype correlation of symptom severity has been described according to the number of polyalanine repeats of PARMs, and NPARMs are generally associated with the most severe symptoms [5, 6].

The increase in CCHS awareness over the last decade has resulted in earlier screening and interventions for patients with CCHS [7]. Therefore, recent studies have focused not only on patient survival but also on neurodevelopment and cognitive skills [8]. In 2017, the Korean Ministry of Health and Welfare designated CCHS as a rare disease, which means that medical care is covered by the national insurance copayment system, and awareness of CCHS has increased. The clinical features, treatment strategies, and outcomes of Korean patients with CCHS have not been previously reported.

We aimed to present a comprehensive single-center experience of patients with CCHS, including key clinical features, treatment strategies, and outcomes. To date, this is the first and largest cohort of patients with CCHS in South Korea.

## Materials and methods

### Study design

**Fig. 1:**
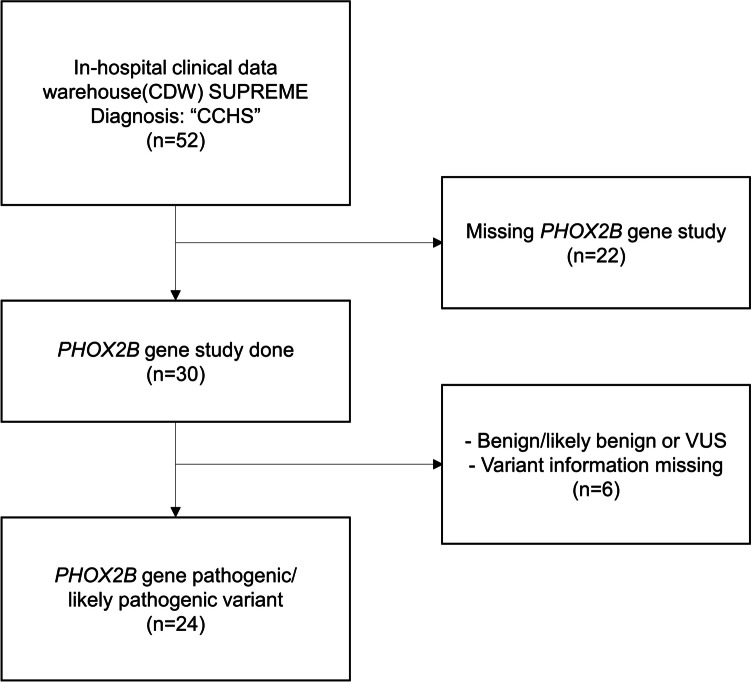
Flowchart of study subjects. *CCHS*, congenital central hypoventilation syndrome; *VUS*, variant of unknown significance

A retrospective chart review was performed on patients diagnosed with CCHS between January 2001 and July 2023 at Seoul National University Hospital (SNUH). First, we screened patients with CCHS diagnostic codes in the in-hospital clinical data warehouse (CDW) SUPREME. Patients who underwent *PHOX2B* genetic analysis were included in this study. Patients with missing variant information and those with benign, likely benign, or variants of uncertain significance (VUS) were excluded (Fig. 1). After collecting demographic data, genotypes, ventilation methods, and clinical features related to autonomic dysfunction, clinical manifestations, and management according to genotype were reviewed. Patients were also categorized into those born before and after CCHS was designated as a rare disease in Korea in June 2017. The clinical characteristics and management were compared between the two groups. Institutional Review Board (IRB) approval (IRB no. 2307-094-1449) was obtained from SNUH for a retrospective observational study with a waiver of informed consent.

### Genetic study

Peripheral blood samples were collected from patients clinically suspected of having CCHS. The 5'UTR coding region and its flanking region of exons 1–3 of the *PHOX2B* (OMIM:603851) gene were analyzed by polymerase chain reaction and direct sequencing, and the deletion/duplication of each exon was examined by MLPA kit P318-A1 (Lot# 1009, MRC Holland, Amsterdam, Netherlands). The reference sequences were NC_000004.11, NM_003924.3, and the basic mutation data were obtained from the Human Gene Mutation Database (http://www.hgmd.cf.ac.uk/ac/). For nucleotide numbering, ATG (initiation codon NM_003924.3) was designated as the first codon in the sequence. The clinical manifestations of each base mutation were determined in accordance with the 2015 recommendations of the American College of Medical Genetics and Genomics [9, 10].

Genotypes of PARM mutations are expressed as ‘20/24 ~ 20/33’, where the first number represents the wild type of 20 polyalanine repeats, and the second number specifies the mutation of 24 to 33 polyalanine repeats.

### Phenotype data

To collect phenotypic data, pertinent data from medical records, including birth weight, gestational age, height, weight, vital signs, ventilator setting records, laboratory data, echocardiography, electrocardiogram, electroencephalogram, radiologic evaluation of the abdomen and brain, surgical records, and medication prescriptions, were thoroughly reviewed. Symptoms related to ANSD were also recorded. The neurodevelopmental outcomes were evaluated by pediatric neurologists and rehabilitation specialists.

### Statistical analysis

Data are presented as number (percentage) or median (interquartile range). The correlation between the genotype and phenotype was analyzed using the Spearman rank correlation test. To compare the clinical features and management of patients born before and after the CCHS rare disease designation, non-parametric testing was performed. Fisher’s exact test was used to compare proportions, and Wilcoxon rank sum test was used to compare medians. Height, weight, and body mass index (BMI) z-scores were calculated according to the 2017 Korean National Growth Charts [11]. The growth parameter z-scores for each age group were compared to 0 using the one-way Wilcoxon rank-sum test. Statistical significance was set at P < 0.05. All statistical analyses were performed using R version 4.3.2 (R Foundation for Statistical Computing, Vienna, Austria).

## Results

### Demographic characteristics of selected subjects

The CDW search for patients with CCHS yielded 52 patients, and after review of *PHOX2B* genetic studies, 20 patients were excluded due to missing gene studies and six patients were excluded due to benign/likely benign variants, VUS, or missing variant information. Twenty-four patients were included in this study (Fig. 1). The study participants’ age ranged from 2 months to 26 years, and the median age at the time of the study was 8 years. Ten patients (41.7%) were male and 17 (70.8%) were born at term, with a median birth weight of 2.873 kg (Table 1). Most patients with CCHS (22 patients, 91.7%) presented with apnea or hypopnea during the neonatal period (within 1 month) and were admitted to the NICU due to respiratory distress, except for two patients who presented symptoms at a later onset (each of them had symptoms at 2 and 36 months after birth). The median age of patients who underwent *PHOX2B* analysis was 2 months. Four patients had a family history of CCHS and were identified as two couples of monozygotic twins (Table 1).

**Table 1:** Clinical characteristics of study subjects

Characteristics of study subjects	(N = 24)
Sex (Male)	10 (41.7%)
Age at clinical manifestations (mo)	0 (0; 0)
Age at genetic diagnosis (mo)	2 (0; 57.75)
Term birth	17 (70.8%)
Birth weight (kg)	2.87 (2.56; 3.27)
Familial history	4 (16.7%)
Home ventilator use	24 (100%)
Tracheostomy	21 (87.5%)
Hirschsprung disease	12 (50.0%)
Arrhythmia	9 (37.5%)
Endocrine dysfunction	7 (29.2%)

Data are presented as number (percentage) and median (interquartile range)

### *PHOX2B* genotypes

The *PHOX2B* genetic study revealed that 22 patients (91.7%) had PARMs and two patients (8.3%) had NPARMs. The most common PARM genotype was 20/27 (11 patients, 45.8%), followed by 20/26 (8 patients, 33.3%). There was one patient each with genotypes 20/25, 20/28, and 20/31.

### Assisted ventilation

All patients used home ventilators, and tracheostomy was performed in 87.5% (21 out of 24) of patients at 1–5 months of age (Table 1). Among the patients who underwent tracheostomy, seven were successfully decannulated at school-age and switched from the T-cannula interface to the nasal or oronasal mask. None of the patients with a high number of GCN repeats (≥ 28) or NPARM underwent decannulation (Table 2).

**Table 2:** Patients with CCHS who underwent tracheostomy and decannulation after tracheostomy

Genotype	Tracheostomy (n = 21)	Decannulation (n = 7)
PARM (n = 22)		
Genotype 20/25 (n = 1)	1 (100%)	0/1 (0.0%)
Genotype 20/26 (n = 8)	8 (100%)	4/8 (50.0%)
Genotype 20/27 (n = 11)	9 (81.8%)	3/9 (33.3%)
Genotype 20/28 (n = 1)	1 (100%)	0/1 (0.0%)
Genotype 20/31 (n = 1)	1 (100%)	0/1 (0.0%)
NPARM (n = 2)	1 (50.0%)	0/1 (0.0%)

All patients started on 24-h ventilator use during the neonatal period; currently, 21 patients (87.5%) use ventilators only during sleep. The three patients who were on full-time ventilation included one infant who was currently 6 months old and two patients who had undergone severe hypoxic ischemic brain damage. Various ventilator modes were used; pressure-support ventilatory (PSV) mode with back up rate for 8 patients (66.7%, 8 out of 24), pressure-controlled synchronized intermittent mandatory ventilation (SIMV) mode for 5 patients (20.8%, 5 out of 24), spontaneous-timed mode for 2 patients (8.3%, 2 out of 24), pressure-controlled (assist control) mode for 4 patients (16.7%, 4 out of 24), and continuous positive airway pressure (CPAP) for one patient.

### Symptoms related to ANSD

**Fig. 2:**
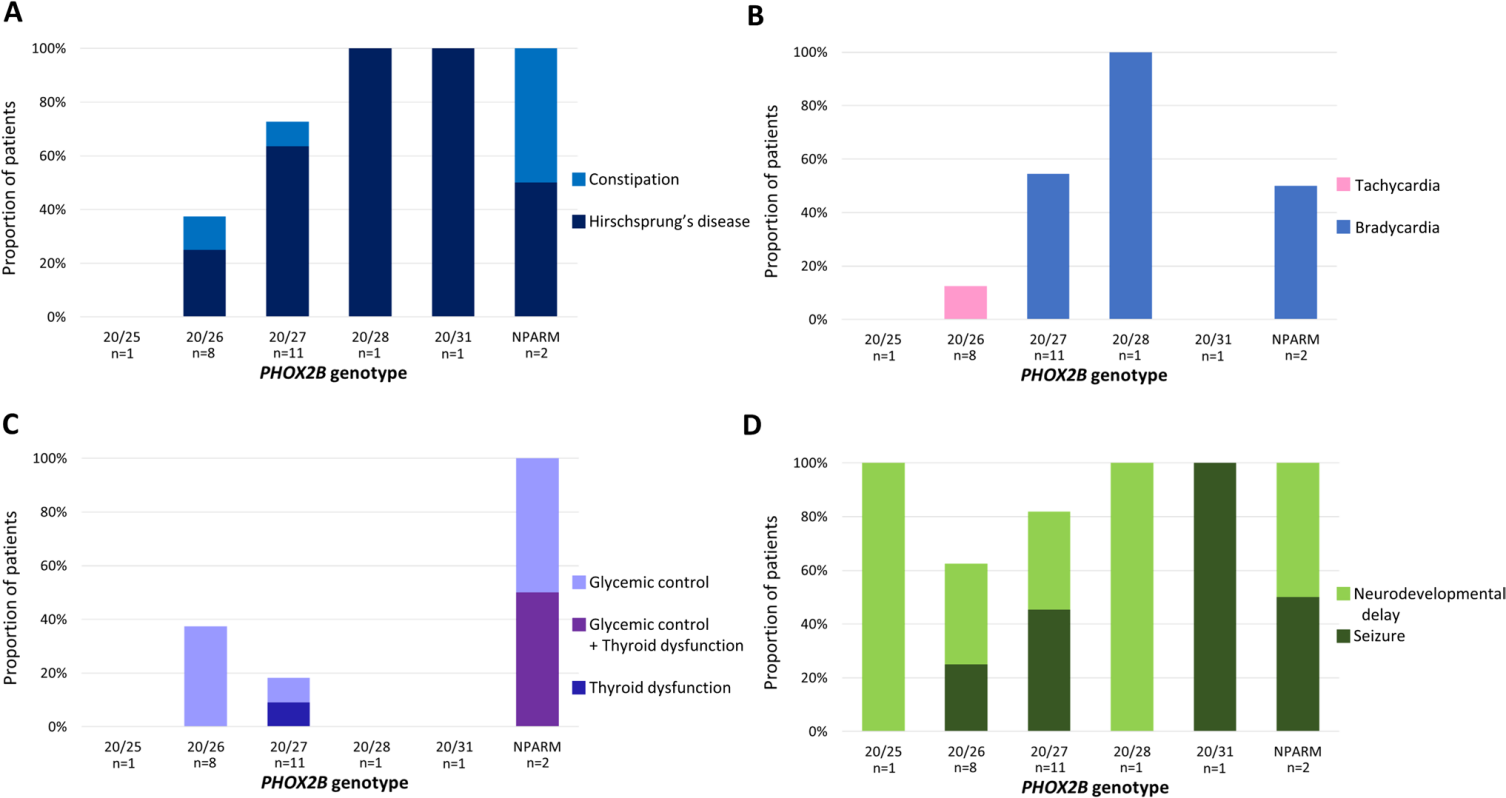
Symptoms related to autosomal nervous system dysfunction. **A**. Gastrointestinal symptoms; **B**. Arrhythmia; **C**. Endocrine dysfunction; **D**. Neurodevelopmental symptoms, *NPARM*, non-polyalanine repeat mutation

Fifteen patients (62.5%) had some degree of constipation requiring treatment. Hirschsprung disease (HD) was diagnosed in twelve patients (50.0%) who underwent a Soave procedure for aganglionosis. All patients with NPARM and genotypes 20/28 and 20/31 had gastrointestinal symptoms, while 72.7%, 37.5%, and 0% had symptoms associated with genotypes 20/27, 20/26, and 20/25, respectively (Fig. 2).

Arrhythmia was present in nine patients (37.5%); one patient had sinus tachycardia and eight patients had sinus bradycardia, of which two patients experienced sinus arrest and were implanted with a loop recorder for electrocardiography monitoring. Half of patients with the PARM genotypes 20/28 and 20/31 had arrhythmias. In total, 57.5% and 12.5% of the patients with genotypes 20/27 and 20/26, respectively, had arrhythmia (Fig. 2).

Endocrine dysfunctions, such as abnormal thyroid function and impaired glycemic control, were also present. Six patients (25.0%) experienced impaired glycemic control with either hyperglycemia or hypoglycemia. One patient was diagnosed with diabetes mellitus and treated with insulin and metformin. Two (16.7%) patients were diagnosed with subclinical hypothyroidism (Fig. 2).

Ophthalmic disorders, including pupillary abnormalities, astigmatism, and strabismus, were also found in seven patients (29.2%). Neuroblastoma was present in one patient with NPARM (4.2%), who underwent excisional biopsy.

### Neurologic features

A wide spectrum of neurological symptoms were prevalent in our cohort. Twenty patients (83.3%) had some degree of developmental delay, including in speech and language development. One patient had ADHD and two had moderate to severe autism spectrum disorder. Nine patients (54.2%) presented with documented seizures and epileptiform discharges on electroencephalography (Fig. 2).

Of the 14 patients with available brain imaging (including MRI and ultrasonography), 57.1% (8 out of 14) had abnormal brain imaging; 62.5% of the patients with abnormal imaging (5 out of 8) had a mild to severe degree of diffuse brain atrophy, four patients (50%) had hypoxic ischemic encephalopathy, and two had intracranial hemorrhage due to hypoxia.

### Genotype–phenotype relationship of *PHOX2B* gene in CCHS

**Fig. 3:**
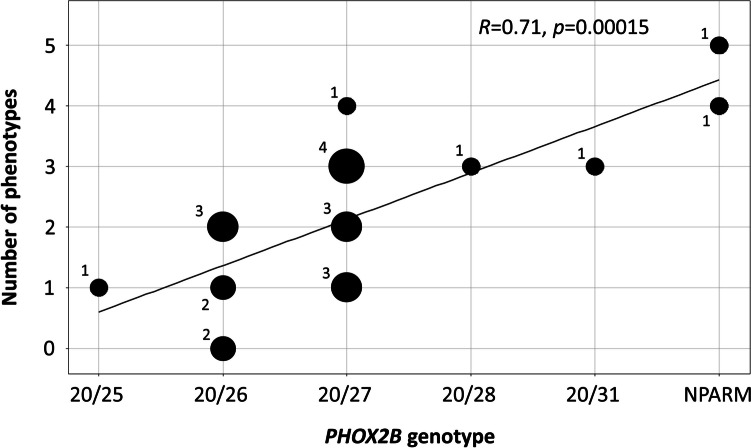
Genotype–phenotype correlation of *PHOX2B* gene in patients with CCHS. Number of phenotypes include ANSD and neurologic symptoms, excluding apnea and hypopnea. Number next to dot indicates number of patients per dot. *R*, Spearman’s Rho; *NPARM*, non-polyalanine repeat mutation

When the number of symptoms, including gastrointestinal symptoms, arrhythmia, endocrine dysfunction, ophthalmologic symptoms, neural crest tumors, and neurocognitive symptoms, were plotted across genotypes, the number of phenotypes ranged from 0 to 5 (Fig. 3). Patients with NPARMs have several clinical manifestations. Patients with fewer GCN repeats tended to exhibit fewer phenotypes. There was a positive correlation between the genotypes in our study, treated as ordinal values, and the number of phenotypes (p < 0.001).

### Growth parameters

**Fig. 4:**
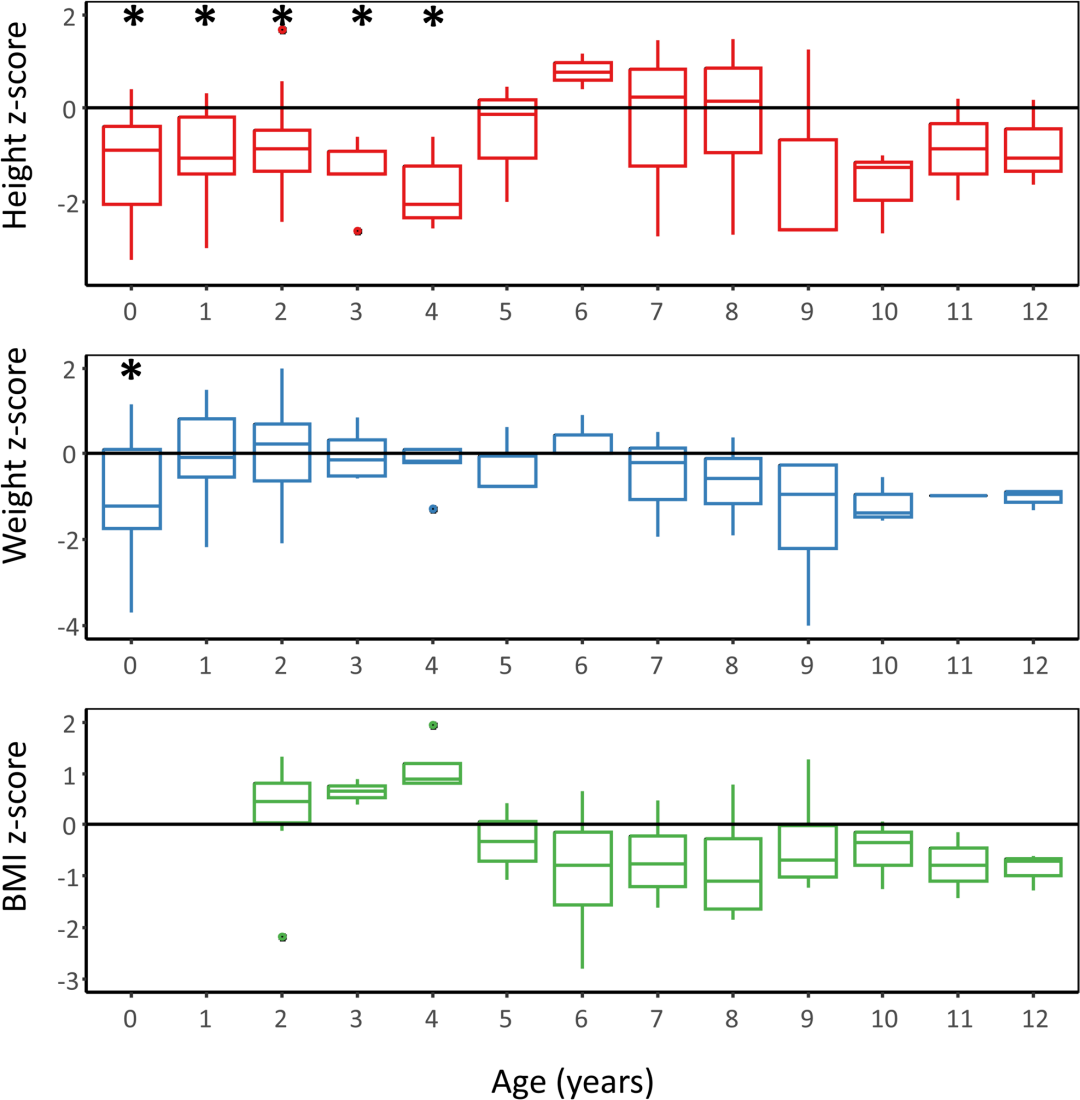
Boxplot of height, weight, and BMI z-scores of CCHS for each age group in years. Asterisk (*) indicates median z-score for age group is significantly smaller than 0. *BMI*, Body mass index

The height and weight of 22 patients were recorded. Eighteen patients had a median height z-score below 0, and four patients (18.2%) had a median height z-score below -2. Twelve patients had a median weight z-score below 0, and two patients (9.1%) had a median weight z-score below -2. Six patients had a median BMI z-score below 0 and no patients had a BMI z-score below -2. Median z-scores for each age group in years were calculated for ages 0–12 years, as we only had three patients who were currently older than 12 years (Fig. 4). The median height z-score was significantly smaller than 0 for patients aged 0–4 years. The median weight z-score was significantly lower than 0 for those aged 0 years. The median BMI z-score did not differ from 0 for any age group.

### Impact of rare disease designation on patients with CCHS

Sixteen patients were born before and eight patients were born after CCHS was designated as a Rare Disease in Korea. There was no difference in age at clinical manifestation, but the age at genetic diagnosis was significantly earlier in patients born after the Rare Disease designation than in those born before. However, the age at tracheostomy and proportion of patients with each phenotype did not differ between groups (Table 3).

**Table 3:** Clinical characteristics of patients before and after CCHS was deemed a Rare Disease in Korea

	**Before (n = 16)**	**After (n = 8)**	***p*-value**
Age at clinical manifestations (mo)	0 (0; 0)	0 (0; 0)	0.338
Age at genetic diagnosis (mo)	2 (1; 79)	0 (0; 2)	0.045
Age of tracheostomy (mo)	2 (1; 4.5)	1 (1; 1.5)	0.356
Constipation	10 (62.5%)	5 (62.5%)	1.000
Hirschsprung disease	8 (50.0%)	4 (50.0%)	1.000
Arrhythmia	6 (37.5%)	3 (37.5%)	1.000
Neurodevelopmental delay	12 (75.0%)	8 (100%)	0.262
Seizure event	5 (31.6%)	4 (50.0%)	0.412

Data are presented as number (percentage) and median (interquartile range)

## Discussion

To our knowledge, this is the first study describing the clinical characteristics and management of patients with CCHS in South Korea. Most patients presented with apnea or hypopnea during the neonatal period, and all were on home ventilators via a T-cannula or mask. Patients had various degrees of ANSD, including gastrointestinal, cardiac, and endocrine dysfunctions, with genotype–phenotype correlations. The height of patients was smaller than that of the normal population for some age groups, whereas weight did not significantly differ. Notably, this is the first study to assess the growth parameters of patients with CCHS. Additionally, with the designation of rare diseases, the genetic diagnosis of these patients has accelerated, emphasizing the need for national support.

Adequate ventilatory support is crucial throughout the lifetime of patients with CCHS [4]. Modes of ventilatory support include positive-pressure ventilation (PPV) via a tracheostomy or mask, phrenic nerve pacing, or negative-pressure ventilation. However, in Korea, phrenic nerve pacing and negative pressure are not commercially available, and all patients are on PPV. Home ventilators are supported by the National Health Insurance System; when physicians prescribe home ventilators, they are rented by home respiratory care companies for approximately $535.00 per month, and 90% to 100% depending on diagnosis and economic status is reimbursed by the government, allowing for affordable mechanical ventilation at home for all patients [12].

Regarding the ventilation interface, tracheostomy is recommended during infancy. For patients with late-onset CCHS who only require nighttime ventilation, mask ventilation is also possible [4, 13]. In our cohort, all but three patients underwent tracheostomy during infancy. In one pair of identical twins, one underwent tracheostomy, but the parents refused tracheostomy for the other twin, even after a diligent explanation of the possible outcomes by the medical staff. In one of the twins receiving mask ventilation, parents had trouble with ventilator compliance during sleep due to child resistance, resulting in frequent desaturation at sleep onset. Currently, a child on a mask ventilator is diagnosed with ASD, while the other twin with a tracheostomy has a mild language developmental delay. It cannot be inferred whether the ventilation interface was the cause of the different neurological outcomes in this pair of twins; however, inadequate ventilation has been associated with adverse neurocognitive outcomes [14]. Decannulation in school-aged children has been reported to be successful, and an algorithm for decannulation has been suggested [15, 16].

Since *PHOX2B* is a transcription factor responsible for neural development during early embryogenesis, alterations in the *PHOX2B* gene result in various ANSDs [5]. When the differentiation of enteric neurons is affected, gastrointestinal manifestations, ranging from constipation to HD, may occur. While the HD rate in CCHS is reported to be approximately 20–31%, [17, 18] 50% of patients were diagnosed with HD in our cohort. Cardiovascular manifestations of CCHS, including transient asystole, sinus bradycardia, and blood pressure abnormalities, can present as frequent syncope and life-threatening sinus pauses, which can lead to many fatal events that eventually require cardiac pacemaker placement [19, 20]. Guidelines recommend yearly screening for arrhythmia with 48 to 72-h Holter monitoring in patients with CCHS [4, 13]. In patients with syncope without sinus pause from Holter monitoring, an implantable loop recorder may be inserted [21]. In our cohort, arrhythmia was present in 37.5% of patients, and 8.3% had sinus pauses. Patients did not have a cardiac pacemaker inserted, but data on the frequency of sinus pauses were collected using implantable loop recorders.

Other manifestations include ocular disorders and neural crest-derived tumors. The most common diseases related to ocular disorders are pupillary abnormalities, convergence insufficiency, strabismus, or ptosis [4]. The prevalence of ocular disorders (29.2%) was lower than reported (46–92%), [22, 23] which may be due to missed ophthalmological screening. Neural crest-derived tumors may occur in 3–5% of patients [4]. Neuroblastomas, ganglioblastomas, and ganglioneuromas are mostly found in PARMs with higher numbers of GCN repeats and NPARMs [24].

Owing to the potential for repeated hypoxic brain damage, neurocognitive functioning is often impaired. Comprehensive neuropsychological assessment is recommended for patients with CCHS to improve their quality of life [8]. In our cohort, almost all patients had some form of developmental delay. However, many patients who were born before 2017, when the diagnosis of CCHS was classified as a rare disease in Korea, did not undergo neurocognitive tests before school age, and most of the older patients' neurocognitive development evaluation had been recently conducted or they were already undergoing treatment after being diagnosed with developmental delay. Younger patients with tracheostomy cannulas inevitably have expressive language delays, which can be resolved once they learn to speak with the tracheostomy. Further testing is needed when children born after the rare disease designation reach school age. Whether neurocognitive impairment or delay is a result of CCHS neuronal development itself or inadequate ventilation is controversial [7, 25]. Ventilation and neurocognitive screening should be provided to minimize potential hypoxic damage.

In the study group, most patients were within the normal range of birth weight; however, at preschool age, their height was below the average height of their peer group. The relationship between mutations in the *PHOX2B* gene and growth impairment is still unknown. However, some studies have reported that growth retardation may be related to ventilator-assisted respiratory failure or hypoxic brain damage. One study found that intermittent hypoxia in rats could impair GH/IGF-1 signaling during the first few weeks of life and might contribute to long-term organ and body growth impairments [26]. We can hypothesize that early hypoxic damage in patients may affect the endocrine system related to growth hormone signaling, resulting in growth retardation.

Since CCHS was classified as a rare disease in Korea in June 2017, patients have been able to receive medical expense and other social welfare benefits. Therefore, patients born after the rare disease designation underwent multidisciplinary follow-up as recommended, but those born before were not able to undergo all recommended screenings. Although genetic diagnosis was accelerated in those born after this classification, there was no significant difference in the prevalence of most phenotypes. We speculate that younger children with milder symptoms were more thoroughly screened after the rare disease designation. However, further studies comparing the two groups are needed when patients born after the classification reach school age.

This study has some limitations. First, this was a single-center study, so not all Korean patients with CCHS were included, but as we are the largest national children’s hospital, this is as close as possible to a nationwide cohort. Second, due to the wide age range and rare disease designation of CCHS, multidisciplinary management for related ANSDs has not been performed thoroughly for older patients. This emphasizes the need for a solid social welfare system for patients with multidisciplinary disorders.

In conclusion, CCHS is a rare disorder with an autosomal dominant mutation in *PHOX2B*, which is characterized by central hypoventilation and accompanied by other diseases related to ANSD. The patients exhibited a spectrum of clinical features, including respiratory, cardiac, endocrine, and other autonomic nervous system-innervated systems. Since all clinical characteristics of CCHS are often not present at birth or upon initial diagnosis and appear in various phenotypes, the management of patients with CCHS requires multidisciplinary care and regular follow-up to ensure optimal outcomes.

## Data Availability

The datasets generated and/or analyzed in the current study are available from the corresponding author upon reasonable request.

## Abbreviations

ANSD: Autonomic nervous system dysregulation
ASD: Autism spectrum disorder
BMI: Body mass index
CCHS: Congenital central hypoventilation syndrome
HD: Hirschsprung disease
NPARM: Non-polyalanine repeat mutations
PARM: Polyalanine repeat mutation
PHOX2B: Paired-like homeobox 2B
VUS: Variants of uncertain significance
