# Correction to: Congenital central hypoventilation syndrome in korea: 20 years of clinical observation and evaluation of the ventilation strategy in a single center

**DOI:** 10.1007/s00431-024-05869-w

**Published:** 2024-11-09

**Authors:** Min Jeong Lee, Ji Soo Park, Kyunghoon Kim, Jung Min Ko, June Dong Park, Dong In Suh

**Affiliations:** 1 Department of Pediatrics, Seoul National University College of Medicine 101, Daehak-Ro, Jongno-Gu, Seoul, 03080 Republic of Korea; 2 Department of Pediatrics, Seoul National University Bundang Hospital, Seongnam, Republic of Korea

**Correction to: European Journal of Pediatrics (2024) 183:3479-3487**

10.1007/s00431-024-05611-6

In the article titled “Congenital central hypoventilation syndrome in Korea: 20 years of clinical observation and evaluation of the ventilation strategy in a single center” published in the *European Journal of Pediatrics*, 183, 3479–3487 (2024) the Institutional Review Board (IRB) number was incorrectly listed.

The incorrect IRB number:

2310-027-1474

The correct IRB number:

2307-094-1449

The article has now been corrected, and the correct IRB number is reflected in the updated version. The authors apologize for the error.

The original article has been corrected.

